# Novel ‘Bacteriospray’ Method Facilitates the Functional Screening of Metagenomic Libraries for Antimicrobial Activity

**DOI:** 10.3390/mps2010004

**Published:** 2019-01-07

**Authors:** Anissa Brahami, Annie Castonguay, Éric Déziel

**Affiliations:** INRS-Institut Armand-Frappier, Laval, QC H7V 1B7, Canada; anissa.brahami@iaf.inrs.ca

**Keywords:** antibiotic, bacterial spray, optimization, library construction

## Abstract

Metagenomic techniques, notably the cloning of environmental DNA (eDNA) into surrogate hosts, have given access to the genome of uncultured bacteria. However, the determination of gene functions based on DNA sequences alone remains a significant challenge. The functional screening of metagenomic libraries represents an interesting approach in the discovery of microbial metabolites. We describe here an optimized screening approach that facilitates the identification of new antimicrobials among large metagenomic libraries. Notably, we report a detailed genomic library construction protocol using *Escherichia coli* DH10B as a surrogate host, and demonstrate how vector/genomic DNA dephosphorylation, ligase inactivation, dialysis of the ligation product and vector/genomic DNA ratio greatly influence clone recovery. Furthermore, we describe the use of an airbrush device to screen *E. coli* metagenomic libraries for their antibacterial activity against *Staphylococcus aureus*, a method we called bacteriospray. This bacterial spraying tool greatly facilitates and improves the functional screening of large genomic libraries, as it conveniently allows the production of a thinner and more uniform layer of target bacteria compared to the commonly used overlay method, resulting in the screening of 5–10 times more clones per agar plate. Using the *Burkholderia thailandensis* E264 genomic DNA as a proof of concept, four clones out of 70,000 inhibited the growth of *S. aureus* and were found to each contain a DNA insert. Analysis of these chromosomic fragments revealed genomic regions never previously reported to be responsible for the production of antimicrobials, nor predicted by bioinformatics tools.

## 1. Introduction

Bacterial resistance to the arsenal of clinically approved antibiotics is currently threatening the efficient treatment and prevention of infectious diseases [1,2,3]. Bacteria are an important source of natural products [4] and their genomes contain biosynthetic gene clusters (BGCs) that are predicted to encode the biosynthesis of interesting compounds that are not yet identified and/or characterized. These clusters might represent pathways for the production of potentially highly active antibiotic compounds and, thus, their identification could have an important positive impact on drug discovery [5]. As only 1% of all microbial species can be cultured using standard laboratory conditions [6], functional metagenomics is considered a key method for the identification of new metabolites, notably via the heterologous expression of DNA in engineered host strains [7]. Using this strategy, bacterial DNA is directly extracted from the environment and cloned in cultured bacterial hosts, providing access to the BGCs present in uncultured bacteria. Several examples of the heterologous expression of BGCs from environmental DNA (eDNA) led to the identification of new metabolites, including terragine from eDNA expressed in *Streptomyces lividans* [8], indolotriptoline from eDNA expressed in *Streptomyces albus* [9] and indirubin from eDNA expressed in *Escherichia coli* [10]. Despite their numerous advantages, functional approaches are very laborious and their reproducibility from one laboratory to another relies on the availability of detailed procedures.

Numerous detailed protocols were previously reported to greatly improve the extraction of eDNA [11,12,13,14,15] and the construction of metagenomic libraries [16,17,18]. Indeed, several factors are believed to influence transformation efficiencies [16] but surprisingly little is known about the actual effect of some of those factors. Consequently, identifying the cause(s) of low transformation efficiencies remains difficult. Factors include (i) metagenomic DNA sampling (which should be representative of the microbial community), (ii) DNA extraction and digestion (which should limit DNA shearing while allowing the efficient recovery of large DNA fragments [19]), (iii) vector-to-genomic DNA ratio, (iv) vector and genomic DNA dephosphorylation, and (v) vector ligation conditions (ligase can be heat-inactivated after ligation and/or ligation product can be dialyzed to eliminate buffer). 

When metagenomics libraries are screened for antibiotic activity, the choice of the method used for the functional screening for clones that produce the smallest zone of growth inhibition is also crucial to the success of the study. The overlay method is the commonly used method for metagenomic library screening [16,20,21]. But, due to the large size of metagenomics libraries to be screened, this method is time-consuming and hinders the discovery of novel antimicrobials by limiting the number of clones to be tested per experiment (in addition to some technical issues, vide infra). Therefore, the development of an efficient and rapid screening method is highly needed in the field in order to promote the use of functional metagenomics approaches for the identification of novel microbial secondary metabolites. 

Herein, we report an optimized and detailed protocol that can be useful for the identification of BGCs from metagenomic libraries using *Burkholderia thailandensis* genomic DNA as a model, by exploiting the heterologous expression of its genetic material into surrogate host *E. coli* DH10B. Moreover, we describe a new method for the formation of target bacteria layers that we named bacteriospray. This technique is more sensitive and allows the screening of larger clone libraries compared to the traditional top agar overlay method. The implementation of the optimized method has led to the construction of a library of size 2.7 × 10^5^ colony forming units (CFU)/μg with a BAC-inserted DNA transformation efficiency of 99%, allowing the screening of 70,000 clones from which four were found to carry an insert providing inhibitory activity against *Staphylococcus aureus*.

## 2. Materials and Methods

### 2.1. Bacterial Strains and Plasmid Growth Conditions

*B. thailandensis* E264 (ATCC 700388, American Type Culture Collection, Manassas, VI, USA) was used for this study. This strain was grown at 30 °C in Lysogeny broth (LB) medium. Electrocompetent *E. coli* strain DH10B used for the cloning and the heterologous expression was purchased from New England Biolabs (NEB), Boston, MA, USA. The library was constructed in the bacterial artificial chromosome pBeloBAC11 (7.5 kb) [22] maintained in *E. coli* K12 ER2420 and can replicate in *E. coli* DH10B with 20 μg/mL chloramphenicol (obtained from NEB). The vector was purified from an overnight LB culture incubated at 37 °C with the Qiagen maxiprep kit. The test strain used for antimicrobial production screening was *Staphylococcus aureus* Newman (laboratory collection), grown at 37 °C in LB medium.

### 2.2. Library Construction Strategies

*B. thailandensis* E264 was grown overnight at 30 °C in LB broth. DNA extraction was done with the FastDNA kit (MP Biomedicals) as recommended by the manufacturer. Briefly, 1.5 mL of bacterial culture was centrifuged, placed in a Lysing Matrix A tube and the appropriate cell lysis solution was added. The mixture was homogenized by vortexing for 30 s (2 times) and DNA extraction was performed as described in the MP protocol. DNA was then partially digested with the restriction enzyme SphI (NEB) for 15 min. The enzyme was then heat inactivated for 15 min at 65 °C. Less than 1 μg of the pBeloBAC11 vector was linearized with one unit of the SphI enzyme (10,000 units/mL) during a 4 h digestion in a 37 °C water bath. Both the vector and the genomic DNA were dephosphorylated when specified (vide infra). One μL of antarctic phosphatase (NEB) was added to every 1 μg of digested DNA reaction mixture with the Antarctic phosphatase buffer (10×), before incubation for 30 min at 37 °C. The enzyme was then heat inactivated at 65 °C for 15 min.

Bacterial cohesive-ends DNA was purified by 0.8% agarose gel electrophoresis prepared with 1× TAE (Tris-Acetate-EDTA) buffer and a DNA size marker. The gel included GelRed stain (Biotium) and DNA was visualized with UV light (260 nm). Outer lanes containing the DNA ladder were cut off from the gel, the position of the desired fragment sizes was marked, and DNA larger than 3 kb was excised [16]. Gel slices were weighted and DNA was extracted with a QIAEX II gel extraction kit (Qiagen). Extracted DNA fragments were ligated overnight to the SphI-linearized pBeloBAC11 vector at 16 °C using NEB T4 DNA ligase. The ligation reaction was optimized by varying the vector and genomic DNA ratio (BAC/gDNA). For that, four BAC/gDNA ratios were tested; 1:3, 1:6, 1:9, 1:12 with 50 ng/μL as a fixed vector concentration (and also at 100 ng/μL in the case of the 1:9 ratio). The DNA ratio was calculated using the following formula:

(1) $$\mathrm{DNA}\;\mathrm{ratio}\;=\frac{\left(\mathrm{ng}\;\mathrm{vector}\;\mathrm{pBeloBAC}11\right)\times (\mathrm{DNA}\;\mathrm{size}\;\mathrm{insert})}{\left(\mathrm{Kb}\;\mathrm{vector}\;\mathrm{size}\right)}\times \left(\frac{\mathrm{BAC}}{\mathrm{gDNA}}\right)$$

The ligase was heat inactivated at 65 °C for 15 min before dialysis for 1 h by adding 20 μL on a millipore mixed cellulose esters (MCE) membrane filter (0.025 μM porosity, VSWP01300) floating on sterile distilled water into a Petri plate. Next, for transformation by electroporation, 2 μL of this dialyzed ligation solution was added to 25 μL *E. coli* DH10B electrocompetent cells, directly transferred to an ice-cold electroporation cuvette (1 mm electrode gap) and electroporated (25 μF, 200 Ω, 2.2 kV). After 1 h incubation in 1 mL NEB 10-beta/stable outgrowth recovery medium, at 37 °C under agitation, 100 μL, 10^−1^, 10^−2^ and 10^−3^ dilutions were spread on LB plates containing 40 μg/mL 5-bromo-4-chloro-indolyl-β-d-thriogalactopyranoside (X-gal), and 20 μg/mL chloramphenicol and then incubated overnight at 37 °C. Briefly, colonies formed from non-recombinant cells appeared blue in color whereas colonies formed from the recombinant ones appeared white. The transformation efficiency (T) was calculated using formula (2) and the average insert size of the library was determined by analyzing different white and blue clones by vector purification followed by SphI digestion and agarose gel electrophoresis.

(2) $$T=\frac{\mathrm{CFU}\;\mathrm{control}\;\mathrm{plate}}{\mathrm{pg}\;\mathrm{BAC}\;\mathrm{DNA}}\times \frac{{1\times 10}^{6}}{\mu g}\times \frac{\mathrm{volume}\;\mathrm{of}\;\mathrm{transformants}}{\mathrm{volume}\;\mathrm{plated}}\times \mathrm{dilution}\;\mathrm{factor}$$

### 2.3. Derivation of a Chloramphenicol Resistant Strain

Since *S. aureus* Newman is unable to grow on 20 μg/mL chloramphenicol, a concentration required for overlay and bacteriospray experiments, a resistant strain was derived by selection on antibiotic gradient LB agar plate. First the Petri dish was tilted to create a slope and 10 mL of LB agar containing 20 μg/mL chloramphenicol was poured. After agar drying, a second 10 mL of LB agar with no antibiotic was added, creating a zero to 20 μg/mL gradient. The inoculation was started from the position of no antibiotic side [23] and a resistant mutant was picked for further experiments. 

### 2.4. Top Agar Overlay Method

The top agar overlay method was used according to Brady (2007). Screening for antimicrobial activity was carried out using the two-layer overlay method with the chloramphenicol-resistant *S. aureus* as the test strain. *E. coli* DH10B clones carrying genomic DNA fragments were grown for 24 h at 37 °C on 20 × 20 mm^2^ square plates containing LB agar with 40 μg/mL (X-gal), 1 mM isopropyl-β-d-thiogalactopyranoside (IPTG) and 20 μg/mL chloramphenicol. The number of *E. coli* colonies per plate was optimized by using different amounts of a 10^−2^ dilution of the initial library to allow detection of very small zones of growth inhibition. Plates were then incubated at room temperature for an additional 5 days to promote the accumulation of metabolites around colonies while preventing their excessive development. Before overlaying the plates with 10 mL LB solidified with 0.75% agar, the *S. aureus* assay strain grown overnight in LB medium was suspended to a final OD_600_ = 0.05 in this top agar cooled at 50 °C. Carefully, the top agar was poured on the top of the plates (so that the *E. coli* colonies would not be disturbed). Plates were incubated overnight at 30 °C for 24 h–48 h and were then visually screened for *E. coli* colonies that produce clear zones of growth inhibition in the *S. aureus* hazy layer.

### 2.5. Bacteriospray Screening Method

**Figure 1:**
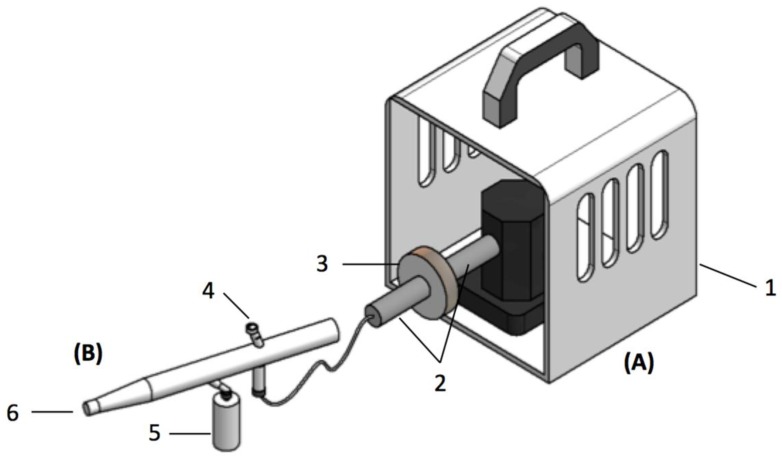
Sketch of the device used to spray a thin layer of *S. aureus* suspension using an airbrush, consisting of the following components: (**A**) an air delivery system, including a compressor (1), a connector (2), a 0.2 μM filter (3); and (**B**) an airbrush, including a needle cap (4), a tank (5), and a nozzle for bacterial suspension mist (6).

As an alternative to the top agar overlay method, an airbrush tool (Anest Iwata-Medea model Revolution BCR, Anest Iwata-Medea, Inc, Portland, OR, USA) was used to spray a mist of bacteria on agar plates containing colonies of the *E. coli* DH10B, transformed with the BAC library prepared as described above (Figure 1). The spray inoculation was performed under a class II laminar flow hood. Plates were placed inside a lidless plastic box to limit aerosols exposure. The airbrush was connected to a compressor delivering air at 20 psi. A Whatman Nylon 0.2 μm filter (L # 3066 Life Sciences, Sigma-Aldrich, ON, Canada) was placed at the outlet of the compressor to avoid any contamination coming from the air source. The airbrush was equipped with a sterile 20 mL plastic tank containing the bacterial suspension. The airbrush needle tip was adjusted so that it would not quite touch the cap, leaving just enough room for air to escape. The airbrush and the tank were decontaminated with ethanol 70% and washed with sterile water before use. 

The screening plates were prepared as above, with an optimized number of clones per plate grown for 24 h. About 5 mL of a 10^5^ *S. aureus* suspension in LB was sprayed as a mist on the plates. Plates were placed vertically at a 60° angle from the spray direction and the airbrush was placed at about 20 cm from the plate. The flow rate was adjusted to obtain a very thin layer of the bacterial suspension, at the eye detection limit. Plates were then incubated at 30 °C for 24 h–48 h before being screened visually, as described above.

### 2.6. Growth Inhibition Zone Confirmation, DNA Sequencing and Insert Analysis 

Clones producing a zone of growth inhibition, presumptive of diffusible antibiotics [24,25,26], were picked, purified onto LB containing 40 μg/mL chloramphenicol and tested again for confirmation of *S. aureus* growth inhibition. Briefly, 5 μL of overnight preculture of purified clones were spotted on LB plates containing 20 μg/mL chloramphenicol and 1 mM IPTG, incubated overnight at 37 °C followed by 5 days at room temperature. The antibacterial activity was then tested using the bacteriospray method, as described above.

Polymerase chain reaction (PCR) amplification of confirmed clones was performed with miniprepped BAC DNA as template using the following primer pairs: M13 F (-40) (5’-GTTTTCCCAGTCACG-3’) and M13 R (-26) r (5’-CAGGAAACAGCTATG-3’). The PCR reaction was performed in a 60 μL reaction volume containing 10 μL of 5X Q5 buffer (NEB), 10 μL of Q5 high GC enhancer 5X, 2.5 μL of 10 pmol/μL of each forward and reverse primers, 1 μL of 0.5 ng DNA template, 1 μL of 10 μM dNTPs and 0.5 μL of Q5 Taq. Reaction conditions consisted of an initial denaturation by heating at 98 °C for 30 s, followed by 30 cycles of denaturation at 98 °C for 10 s, annealing at 57 °C for 30 s, extension at 72 °C for 1 min, and final extension at 72 °C for 5 min. Amplified products were visualized by 1% agarose gel electrophoresis. Amplicons were dialyzed as described above before Sanger DNA sequencing (Institut de recherches cliniques de Montréal; IRCM). Sequence analysis was done using the CLC sequence viewer (version 7). Since the genome of *B. thailandensis* E264 was previously sequenced (with data available from the www.burkholderia.com website), the exact nature of cloned genes could be identified using bioinformatics tools. Sequence-based homology searches in the GenBank database were carried out using the protein BLAST (BLASTp) program (BLOSUM62, existence: 11, extension: 1, conditional compositional score matrix adjustment). 

### 2.7. Statistical Analysis

Each experiment was performed at least in triplicates (n = 3) and experimental values are presented as means +/− standard deviation. Statistical analysis was done using Prism 6 (GraphPad Prism version 6.00, GraphPad Software, Inc, La Jolla CA, USA). A one-way analysis of variance followed by a Tukey’s post hoc test for multiple comparison groups were computed to evaluate the effect of dialysis, x, y, on transformation efficiency (α < 0.05). As an alternative, a Kruskal–Wallis test followed by a Dunn’s post hoc test for multiple comparison groups were used to compare transformation efficiency as a function of ligation product dialysis due to unequal variance between the three different treatment groups. 

## 3. Results 

### 3.1. Transformation Efficiency Optimization

**Figure 2:**
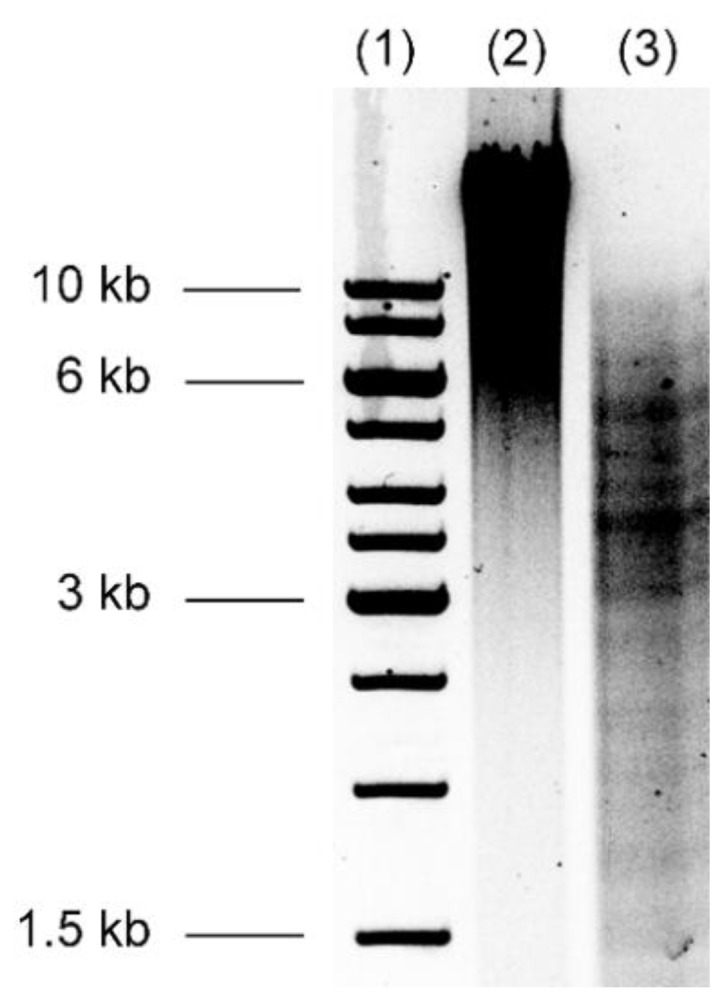
*Burkholderia* DNA size after SphI digestion. 0.8% agarose gel loaded with Thermo Scientific GeneRuler 1 kb DNA Ladder molecular weight standard (lane 1), *B. thailandensis* DNA extraction sample (lane 2) and genomic DNA following SphI digestion sample (lane 3).

DNA fragments between 0.1–10 kb were obtained after SphI digestion of the extracted *B. thailandensis* E264 DNA (Figure 2). Fragments larger than 3 kb were purified and used for all cloning experiments involving pBeloBAC11 as the expression vector and *E. coli* DH10B as the surrogate host.

**Figure 3:**
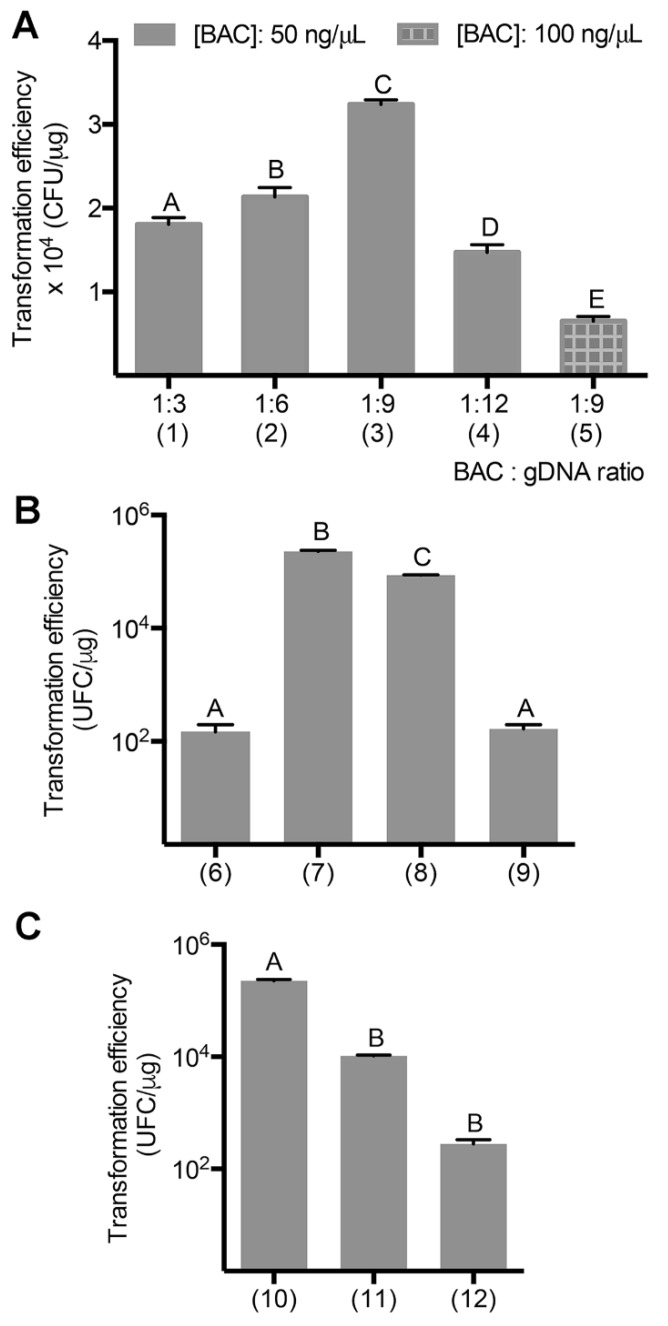
Influence of various parameters on the transformation efficiency of pBeloBAC11 in *E. coli* DH10B. (**A**) Effect of vector to genomic DNA ratio (BAC:gDNA) and vector concentration (ng/μL): (1), (2), (3), (4) DNA and vector ratio from 1:3 to 1:12 respectively under 50 ng/μL vector concentration; (5) DNA and vector ratio 1:9 under 100 ng/μL vector concentration (conditions: vector DNA was dephosphorylated before ligation and ligation product underwent dialysis). (**B**) Effect of vector (BAC) and genomic DNA (gDNA) dephosphorylation on the transformation efficiency: (6) BAC and gDNA not dephosphorylated; (7) BAC and gDNA dephosphorylated; (8) BAC dephosphorylated and gDNA not; (9) gDNA dephosphorylated and BAC not (conditions: 1:9 BAC:gDNA ratio was used, ligase was inactivated and ligation product underwent dialysis). (**C**) Effect of ligase inactivation and dialysis of the ligation product: (10) ligase inactivation and dialysis; (11) dialysis only; (12) ligase inactivation only (conditions: 1:9 BAC:gDNA ratio was used, BAC and gDNA were dephosphorylated). Error bars indicate the standard deviation of three independent experiments, and statistical significance was determined using Tukey post hoc test. A, B, C, D and E represent Tukey’s multiple comparison groups (α = 0.05).

Optimization of the cloning efficiency was performed by varying several parameters, starting with the vector to genomic DNA ratio (BAC/gDNA). Surprisingly, varying digested DNA ratio from 1:3 to 1:12 did not have a considerable impact on the resulting transformation efficiency (Figure 3A columns (1), (2), (3), (4)). Nevertheless, best results were obtained when a BAC/gDNA ratio of 1:9 was used (3.2 × 10^4^ CFU/µg). It is noteworthy that increasing the BAC vector DNA concentration from 50 ng/μL to 100 ng/μL led to a 5-fold decrease in transformation efficiency (Figure 3A column (5)). Thus, for all further optimization experiments, a 1:9 BAC/gDNA ratio and a vector DNA concentration of 50 ng/μL were selected.

The importance of vector dephosphorylation was then assessed, a step that was already integrated in the protocol, prior to the ligation. As previously reported for other systems [27] this parameter was found to have a significant influence on the cloning efficiency. When 5’-phosphate groups of the vector DNA were not removed, a 1000-fold decrease (from 10^5^ to 10^2^ CFU/µg) in cloning efficiency was observed (Figure 3B). The impact of the dephosphorylation of the genomic DNA was also investigated. In contrast to the vector DNA, the dephosphorylation of the genomic DNA alone was found to lead only to a small increase in transformation efficiency (from 8.6 × 10^4^ to 2.2 × 10^5^ CFU/µg). Interestingly, under those conditions, no blue colonies were observed even after an overnight incubation at 4 °C compared to 10% blue colonies when genomic DNA was not dephosphorylated. To further confirm that the maximum transformation efficiency was achieved, the effect of the dialysis of the ligation product and of ligase inactivation on the transformation efficiency were investigated. These two steps were found to be important for the efficiency of the protocol reported here, the dialysis of the ligation product being even more influential. Omission of dialysis of the ligation product or inactivation of the ligase before dialysis led to a 1000-fold and a 10-fold decrease in transformation efficiency, respectively (Figure 3C). 

### 3.2. Burkholderia thailandensis Library Construction

**Figure 4:**
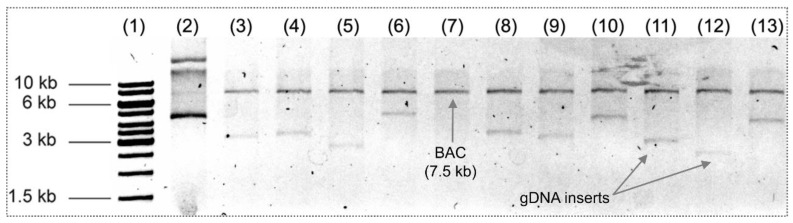
Distribution of inserts sizes of bacterial artificial chromosome (BAC) clones. 1% agarose gel loaded with Thermo Scientific GeneRuler 1 kb DNA Ladder molecular weight standard (lane 1), pBeloBAC11 DNA not digested sample (lane 2), pBeloBAC11 DNA control sample digested with SphI (7.5 kb) (lane 7), confirmation of the presence of a DNA insert from the B. thailandensis genome (lanes 3–6 and 8–13).

Several independent libraries were constructed in the pBeloBAC11 vector using genomic DNA from *B. thailandensis* E264 for zone of growth inhibition screening. The best transformation efficiency was obtained under the optimized conditions described previously: BAC/gDNA ratio of 1:9, dephosphorylation of vector DNA (50 ng/μL), dephosphorylation of genomic DNA, T4 ligase inactivation and dialysis of the ligation product. The best transformation efficiency obtained was 2.4 × 10^5^ ± 0.2 CFU/μg. SphI enzyme-assisted restriction digestion of 12 random clones demonstrated that insert DNA size varied between 2–5 kb (Figure 4).

### 3.3. Top Agar Overlay vs Bacteriospray Assay for the Functional Screening of Metagenomic Libraries

**Figure 5:**
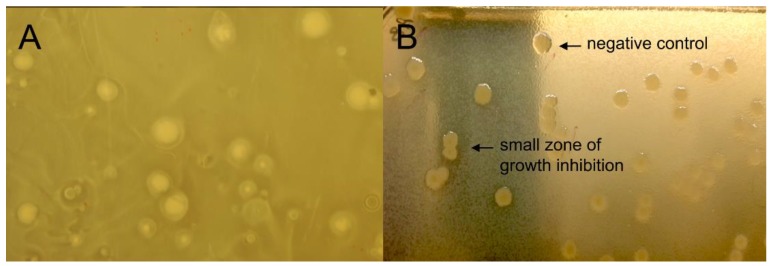
Examples of growth inhibition zones observed from antibiotic-producing clones from *E. coli* library. (**A**) the top agar overlay method vs (**B**) the airbrush assay.

**Figure 6:**
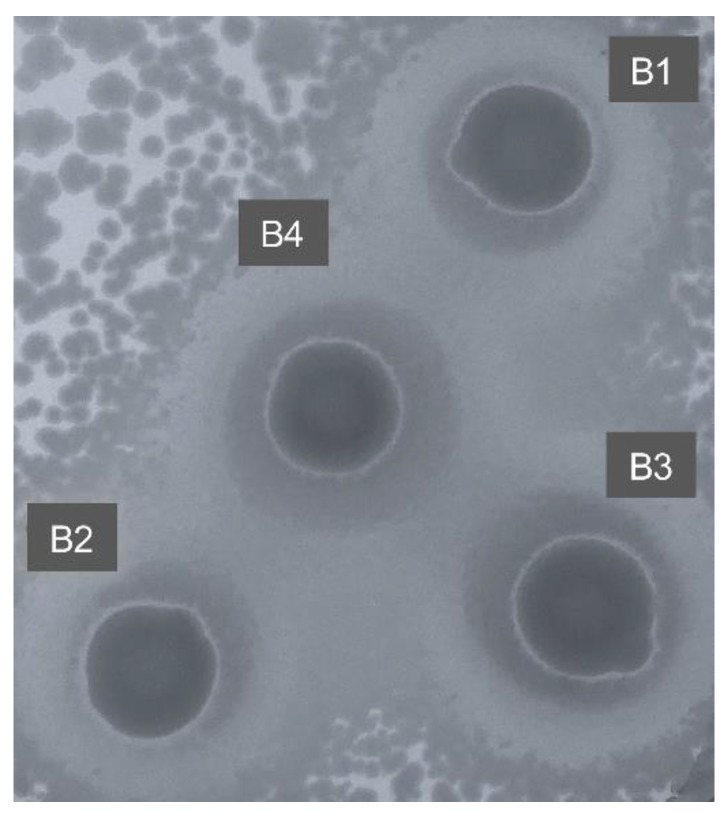
Confirmation of the antibacterial activity of four positive *E. coli* clones B1–B4 against *S. aureus*, using the airbrush assay.

To investigate the functional diversity of the genomic DNA libraries constructed, a distribution of the clones was carried out in 20 × 20 mm^2^ LB square plates. The first method used for antimicrobial screening was the traditional top agar overlay method. Using the method, we could accommodate a maximum of 300–500 clones per plates. In our experience, *E. coli* colonies were disturbed when the top agar layer containing *S. aureus* bacterial suspension was poured (Figure 5A), causing a non-homogenous top agar suspension layer and colonies streaking. Moreover, due to this lack of homogeneity of the resulting bacterial layer, the visual screening for small zones of growth inhibition was very difficult, leading to ambiguous results. To overcome the aforementioned well-known problems, we therefore developed a novel bacteriospray method for the functional screening of genomic libraries (Figure 5B). A pilot study was performed to optimize the airbrush suspension volume to be used, as well as to ensure no contaminant would come from the air (data not shown). The most adequate airbrush spraying volume was estimated to be 5 mL of *S. aureus* bacterial suspension for eight 20 × 20 mm^2^ square plates. The *S. aureus* suspension was sprayed as a mist to obtain a very thin bacterial layer. A pressure of 20 psi was found to be optimal. Whereas 10 psi were not sufficient to properly disperse the bacteria, 30 psi were too strong, causing an unstable air flow and consequently an heterogenous bacterial film. As a pressure of 20 psi allowed proper bacterial growth after using the spraying tool, possible cell damage was assumed to be minimal. Notably, using this method, each plate could accommodate up to 7000 clones. The bacteriospray method was found to be more efficient and sensitive than the top agar overlay method. Using this strategy, 70,000 clones were screened for antibacterial activity, and four clones (B1–B4) led to the formation of a small zone of growth inhibition. Finally, their activity against *S. aureus* was confirmed, as shown in Figure 6.

### 3.4. Sequence Analysis of Inserted Genes 

**Figure 7:**
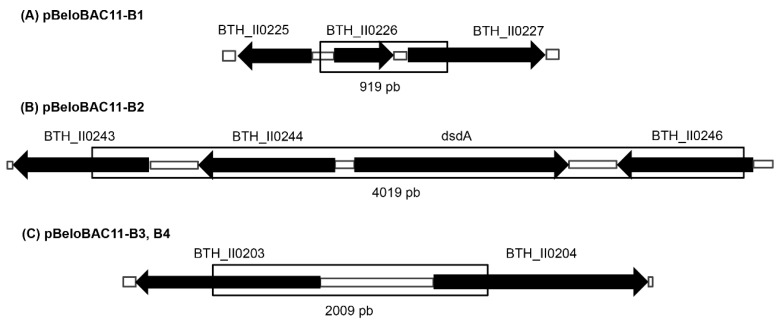
Chromosomal context of the DNA insert identified in the four selected clones derived from *B. thailandensis* E264 chromosome II. Inserted region of the clone (**A**) pBeloBAC11-B1, (**B**) pBeloBAC11-B2 and (**C**) pBeloBAC11-B3, B4.

Following PCR amplification, sequencing of the DNA contained in the four active clones B1–B4 was achieved. By comparison with the whole *B. thailandensis* E264 genome, the insert sequence of each clone was identified (Figure 7). Two of them were found to be identical (B3 and B4) but, importantly, none of them were previously reported to be responsible for the production of an antibiotic, nor predicted by bioinformatics tools (Table 1). 

## 4. Discussion

In this study, we developed and optimized a method to extract and clone DNA fragments to construct and efficiently screen a genomic library for antimicrobial activity in a surrogate host, here *E. coli* DH10B. This functional genomic approach allowed the identification of gene functions that could not have been predicted from DNA sequences alone [28], or that might not have been expressed under standard culture conditions [29]. The pBeloBAC11 vector was selected for this investigation, as it includes a T7 promoter that allows the use of the highly effective T7 RNApol, facilitating the strong transcription of foreign DNA inserts [30] and consequently increasing the probability of detecting clones expressing antibacterial activity. Moreover, this vector can accommodate large DNA inserts, making the developed method suitable for the screening of very large size metagenomics libraries, allowing a reduced number of clones to be tested while increasing the probability of identifying new bioactive molecules for which large BGC are used for their biosynthesis [31,32]. 

Dephosphorylating both the vector and the DNA insert resulted in virtually no blue colonies after vector transformation, indicating the absence of empty vectors (and that the highest transformation efficiency was achieved). Besides BACs, plasmid and cosmid vectors can also be used with this protocol. The choice of vector is based on several criteria, including the host bacteria, the expected size of DNA inserts, the type of function targeted by the screening and the screening strategy. Large-insert libraries are required to recover large gene clusters coding a complex pathway [8]. However, small-insert libraries can be employed for the identification of novel molecules encoded by a single gene or small operon. Here, the size of DNA fragments inserted in the clones that expressed antimicrobial activity varied from 0.9 to 4 kb. Since the size of digested fragments did not exceed 10 kb (Figure 2), it was not possible to construct a larger insert library. Whereas this insert size is large enough to isolate antimicrobial activities encoded by small operons, it is not sufficiently large for the recovery of large gene clusters [33]. 

Sequencing the selected clones revealed that two were identical (B3 and B4) and that gene BTH_II0204 was included in the insert. Curiously, this gene is part of a four gene operon predicted to contain a single nonribosomal peptide synthetase (NRPS) gene BTH_II0204-0207 in the *B. thailandensis* E264 and *B. pseudomallei* K96243 genomes [34,35]. These genes are annotated as NRPS, a dehydratase, an isomerase, and a methyltransferase [34]. Since the nature of the complete operon is unknown, it was not possible to conclude on the origin of the inhibitory activity. Clone B1 contains the gene BTH_II0226 that is predicted to encode a Snoal-like protein in *B. thailandensis* E264. These proteins encode small polyketides cyclases, which catalyze the ring closure step in the biosynthesis of polyketide antibiotics produced in *Streptomyces* [36], but the function is not determined in *B. thailandensis*. It is noteworthy that a search was also performed with the BAGEL database [37], which suggested a possibility for the three distinct DNA sequences to be responsible for the production of an antimicrobial peptide. Further analysis and plasmid gene subcloning of these clones will be pursued in order to identify the origin of the observed antimicrobial activity.

This study also highlights that the success/failure of a functional genomic study depends significantly on the phenotype screening method used, a typically overlooked aspect of this promising strategy. The use of the bacteriospray method has led to significantly enhanced screening efficiencies compared to the traditional top agar overlay method, which causes the lack of top agar homogenous layer and colonies streaking, making identification of the active clones difficult. For instance, with this newly developed method, an average of 5000–7000 clones per plate could be tested at a time, compared to a maximum of 300–500 clones when the traditional top agar overlay method was used. Our results are in agreement with other genomic functional screening efforts for which a maximum of 500 to 1500 colonies per plate could be screened using the overlay method [10,16,21], making the bacteriospray method approximately 5–10 times more efficient for the screening of large-size genomic libraries. A similar method was previously used for spraying monolayers of *Bacillus subtilis* spores [38,39,40], but to our knowledge, this technique was never used to spray live bacteria. The spraying of a bacterial suspension provides a homogenous monolayer lawn of bacteria that allows the sensitive detection of the thinnest bacterial inhibition zones, which will greatly benefit functional metagenomic studies. 

## 5. Conclusions

In this article, we described an improved protocol for the construction and screening of functional metagenomic libraries. We also developed a new bacterial spraying method which will be useful for the screening of large libraries. We demonstrated that this new tool allows the identification of small zones of growth inhibition and is effective and sensitive enough to be used for the screening of eDNA metagenomic libraries. We believe this protocol could play an important role in the discovery of novel antibiotics. 

## Figures and Tables

**Table 1:** Locus tag identified in the four clones derived from *B. thailandensis* E264 chromosome II metagenomic library in pBeloBAC11 vector.

**Clone Locus Tags**	**Similar Protein (Accession Number)**
pBeloBAC11 B1	
*BTH_II0226*	Snoal-like protein (3H3H A)
*BTH_II0227*	Conserved hypothetical protein (ABC35626.1)
pBeloBAC11 B2	
*BTH_II0243*	LysR family transcriptional regulator (WP_009894988.1)
*BTH_II0244*	LysR family transcriptional regulator (WP_009894990.1)
*BTH_II0245*	*dsdA* D-serindeshydratase (Q2T8Q2.1)
*BTH_II0246*	LysR family transcriptional regulator (WP_011400849.1)
pBeloBAC11 B3 & B4	
*BTH_II0203*	Carbohydrate diacid regulator (ABC35448.1)
*BTH_II0204*	Peptide synthase, putative (ABC34379.1)
